# Systemic Oxidative Stress, Aging and the Risk of Cardiovascular Events in the General Female Population

**DOI:** 10.3389/fcvm.2021.630543

**Published:** 2021-02-09

**Authors:** Martin F. Bourgonje, Arno R. Bourgonje, Amaal E. Abdulle, Lyanne M. Kieneker, Sacha la Bastide-van Gemert, Ron T. Gansevoort, Stephan J. L. Bakker, Douwe J. Mulder, Andreas Pasch, Jumana Saleh, Sanne J. Gordijn, Harry van Goor

**Affiliations:** ^1^Department of Pathology and Medical Biology, University Medical Center Groningen, University of Groningen, Groningen, Netherlands; ^2^Department of Gastroenterology and Hepatology, University Medical Center Groningen, University of Groningen, Groningen, Netherlands; ^3^Division of Vascular Medicine, Department of Internal Medicine, University Medical Center Groningen, University of Groningen, Groningen, Netherlands; ^4^Division of Nephrology, Department of Internal Medicine, University Medical Center Groningen, University of Groningen, Groningen, Netherlands; ^5^Department of Epidemiology, University Medical Center Groningen, University of Groningen, Groningen, Netherlands; ^6^Department of Physiology and Pathophysiology, Institute for Physiology and Pathophysiology, Johannes Kepler University Linz, Linz, Austria; ^7^Department of Biochemistry, College of Medicine & Health Sciences, Sultan Qaboos University, Muscat, Oman; ^8^Department of Obstetrics and Gynecology, University Medical Center Groningen, University of Groningen, Groningen, Netherlands

**Keywords:** menopause, oxidative stress, free thiols, cardiovascular events, population study

## Abstract

**Introduction:** Menopause is associated with increased cardiovascular risk, in which oxidative stress plays a pivotal role. Systemic oxidative stress is reflected by decreased levels of free thiols (R-SH, sulfhydryl groups), which are key components of the extracellular antioxidant machinery. In this study, we investigated the relation between serum free thiols as marker of oxidative stress and the female cardiovascular phenotype, as well as potential associations with the risk of cardiovascular (CV) events in pre- and postmenopausal women from the general population.

**Methods:** Female participants (*n* = 2,980) of the Prevention of REnal and Vascular ENd-stage Disease (PREVEND) cohort study were included. Serum free thiol concentrations were analyzed for associations with demographic, clinical, biochemical, and gynecological parameters, as well as with menopausal status and, prospectively, with the risk of CV events.

**Results:** Postmenopausal women had significantly reduced levels of serum free thiols (4.8 ± 1.0 vs. 5.2 ± 1.0 μmol/g, *P* < 0.001) compared to reproductive women. In multivariable analyses, serum free thiols were significantly associated with menopausal status (OR 0.70 [0.49–0.98], *P* = 0.039), even when adjusted for potential confounding factors, except for age (*P* = 0.550). Prospectively, serum free thiols were significantly associated with the risk of CV events (HR 0.52 [0.27–0.97], *P* = 0.040), even with covariate adjustment, although this disappeared when correcting for age.

**Conclusion:** In this study, we revealed serum free thiols to be strongly associated with the female cardiovascular phenotype as well as with female risk of CV events, where the influence of age itself seemed to outweigh that of female menopause. Future studies are warranted to further unravel the clinical utility of serum free thiol levels in the context of female cardiovascular risk management.

## Introduction

The menopause marks the permanent end of ovarian follicular activity and thereby the end of women's menstrual cycles. It is associated with a sudden increase in risk of cardiovascular events (1). It is evident that the changing hormone levels of estrogen and progesterone are involved in this process as typically estrogen levels lower after the menopause (2). The menopause is also thought to induce oxidative stress. This may in part be caused by reduced estrogen production, which is known to exert antioxidant effects (3).

Reactive oxygen species (ROS) are of crucial biological value in various physiological systems including the regulation of immunity, autophagy, differentiation and longevity (4). They are also important in the response to hypoxia. Oxidative stress however is characterized by an aberrant production of ROS often coinciding with a decreased availability of antioxidants that neutralize these reactive species. It is a key pathophysiological mechanism in many inflammatory and hypoxic conditions (5, 6). Oxidative stress leads to accumulating damage at all levels of biological organization, and is closely associated with systemic inflammation (7).

Serum free thiol groups (sulfhydryl groups, R-SH) are considered to be a representative systemic biomarker of local and systemic oxidative stress (8). They are central components of the extracellular antioxidant machinery and possess potent antioxidant activity (9). Free thiols are mainly embedded in circulating cysteine-based proteins, of which albumin is most abundantly present in blood (10). In addition, there are also low-molecular-weight (LMW) free thiols, e.g., homocysteine and glutathione. The sum of non-oxidized protein thiols and LMW thiols is termed *total free thiols*, comprising a dynamic physiological entity and acting as multimodal redox relays by transducing kinetically controlled intra- and extracellular redox exchange reactions (11). Reduced levels of serum free thiols arise from rapid oxidation by high amounts of reactive species (12). Conversely, higher concentrations of free thiols are indicative of a more favorable redox status. In multiple conditions, the systemic redox status as measured by serum free thiols has shown to associate with disease severity and to predict clinical outcome (4, 8, 12, 13). Similarly, reduction of extracellular free thiols is associated with many cardiovascular risk factors, including aging, obesity, smoking, and alcohol consumption (4, 12).

As menopause triggers oxidative stress, which in turn compromises cardiovascular function, the question arises whether serum free thiols, as a systemic marker of oxidative stress, are associated with clinical and biochemical characteristics of menopause in females from the general population. Therefore, we aimed to investigate if serum free thiols could be used to directly and accurately predict the risk of cardiovascular events in pre- and postmenopausal women from the general population. For that purpose, we studied the relation between serum free thiols as marker of oxidative stress and the female cardiovascular phenotype, as well as its potential association with the risk of cardiovascular events.

## Materials and Methods

### Study Population

The PREVEND (Prevention of REnal and Vascular ENd-stage Disease) study is a large-scale, prospective cohort study executed in Groningen, the Netherlands (14, 15). It was started in 1997 and was designed to investigate the relation between urinary albumin excretion and renal and cardiovascular disease. It features data on a large number of variables from inhabitants of Groningen aged 28–75 years. In total, 85,421 subjects were sent a questionnaire and morning urine collection vial by post, to which 40,856 people replied. The questionnaire included questions about demographics, cardiovascular disease, pregnancy and medication. Excluded were participants who were pregnant, diagnosed with diabetes mellitus type 1, or insulin-treated diabetes mellitus type 2. Patients with urinary albumin concentrations ≥10 mg/L (*n* = 6,000) were invited to visit the research clinic of the University Medical Center Groningen (UMCG). As a control group, a randomly selected group of participants with urinary albumin concentrations below 10 mg/L (*n* = 2,592) were also invited to visit the research clinic, for a combined total of 8,592 participants to complete the full PREVEND study program. A second visit between 2001 and 2003 was initiated to collect another set of serum samples from 6,136 of these participants. For this study, serum free thiol levels from pre-and post-menopausal women have been extracted from the PREVEND study (second visit). The total number of women was *n* = 2,980 with known serum free thiol levels and known menstruation status (*n* = 58 without free thiol status, which were excluded). The two main groups were of comparable size (*n* = 1,469 for premenopausal women, *n* = 1,511 for postmenopausal women). This study was approved by the Institutional Review Board (IRB) (full name in Dutch: “Medisch Ethische Toetsingscommissie,” METc) of the UMCG and conducted in accordance with the principles of the Declaration of Helsinki (2013). All study participants provided written informed consent.

### Data Collection

All patients completed a questionnaire containing information about demographics, health status, history of cardiovascular disease, lifestyle habits, and medication use, and anthropometric measurements were performed. Blood pressure was measured automatically every minute for a period of 8 min in supine position (Dinamap XL Model 9300 series device, Johnson & Johnson Medical, Tampa, FL). Blood pressure was defined as the average of the last two measurements. Alcohol usage could be answered with “no,” “1–4 per month,” “2–7 per week,” “1–3 per day,” or “>4 per day.” For smoking there was a distinction between “never,” “former,” and “current.” Waist circumference was measured on bare skin at the natural indentation between the 10th rib and the iliac crest.

### Laboratory Measurements

High-sensitive C-reactive protein (hs-CRP) was measured by nephelometry (Dade Behring Diagnostics, Marburg, Germany). Serum total cholesterol and glucose were measured by dry chemistry (Eastman Kodak, Rochester, NY, USA). High-density lipoprotein (HDL) and low-density lipoprotein (LDL) cholesterol were determined by the Friedewald formula. Triglycerides were measured with an enzymatic method. Serum creatinine was measured enzymatically (Roche Modular, Roche Diagnostics, Mannheim, Germany). Serum alanine aminotransferase (ALT) and aspartate aminotransferase (AST) were measured using the standardized kinetic method with pyridoxal phosphate activation (Roche Modular P, Roche Diagnostics). Serum cystatin C was measured using the Gentian Cystatin C Immunoassay (Gentian AS, Moss, Norway) on a modular analyzer (Roche Diagnostics). Standards provided by the manufacturer were used to calibrate cystatin C (according to the International Federation of Clinical Chemistry Working Group for Standardization of Serum Cystatin C) (16).

### Study Outcomes and Definitions

Fatal and non-fatal cardiovascular events (CV) were considered primary endpoints of the study. Cardiovascular events were regarded as a composite outcome including acute myocardial infarction (AMI), ischemic heart disease (IHD), coronary artery bypass grafting (CABG), percutaneous transluminal coronary angioplasty (PTCA), intracranial hemorrhages, stenosis or occlusion of pre-cerebral or cerebral arteries, and vascular interventions such as aortic peripheral bypass surgery, percutaneous transluminal femoral angioplasty, or carotid desobstruction). All these outcomes were retrieved from the Dutch National Registry of all hospital discharge diagnoses (Prismant). This information was classified in accordance with the International Statistical Classification of Diseases (ICD-10) and the International Classification of Health Interventions (17). Menopause was defined as the self-reported absence of a regular menstruation cycle for a minimum duration of at least 1 year. Hypercholesterolemia was defined as a serum total cholesterol level of >6.5 mmol/L, the use of lipid-lowering drugs or a serum HDL-cholesterol level <0.9 mmol/L. Diabetes mellitus type 2 was defined as a fasting glucose level ≥7.0 mmol/L or the use of oral antidiabetics according to the American Diabetes Association (ADA) guidelines. Estimated glomerular filtration rates (eGFR) were calculated using the combined creatinine cystatin C-based Chronic Kidney Disease Epidemiology Collaboration (CKD-EPI) equation (18).

### Measurements of Serum Free Thiol Concentrations

Serum samples were stored at −80°C until analysis to avoid any significant changes in free thiol stability. Serum free thiol concentrations were measured after applying minor modifications (19, 20). After thawing, serum samples were diluted 4-fold using 0.1 mol/L Tris buffer (pH 8.2). Freezing and thawing does not cause any auto-oxidation processes that could jeopardize our measurements. Using the Varioskan microplate reader (Thermo Scientific, Breda, the Netherlands), background absorption was measured at 412 nm, together with a reference measurement at 630 nm. Following this, 20 μL 1.9 mmol/L 5,5′-dithio-bis(2-nitrobenzoic acid) (DTNB, Ellman's Reagent, CAS-number 69-78-3, Sigma Aldrich Corporation) in 0.1 M phosphate buffer (pH 7.0) was added to the samples and absorbance was measured again after the samples were incubated for 20 min at room temperature. Final concentrations of serum free thiols were established by parallel measurement of an L-cysteine (CAS-number 52-90-4, Fluka Biochemika) calibration curve (concentration range from 15.625 to 1,000 μmol/L) in 0.1 M Tris/10 mM EDTA (pH 8.2). Intra- and interday coefficients of variation (CV) of all measurement values were below 10%. Lastly, serum free thiol concentrations were adjusted to total serum protein levels (measured according to standard procedures) by calculating the free thiol/total protein ratio (μmol/g of protein). This adjustment was performed as serum proteins harbor the largest amount of free thiols and therefore largely determine the amount of potentially detectable free thiols (9).

### Statistical Analysis

Baseline demographics and clinical characteristics were presented using mean ± standard deviation (SD), median [interquartile range, IQR] or proportions *n* with corresponding percentages (%). Assessment of normality was performed using normal probability plots (Q-Q plots) and histograms. Differences between groups for continuously distributed variables were tested using one-way analysis of variance (ANOVA) or Kruskal-Wallis tests, while for categorical variables chi-square tests were performed, as appropriate. Univariable and multivariable linear regression analyses were performed to identify factors that independently associated with serum free thiol levels. From linear regression analyses, standardized beta (β) coefficients and corresponding *P*-values were reported to indicate the strength and statistical significance of the associations. Standardized β-coefficients represent the difference in serum free thiol levels per 1-SD increment for continuous variables and the difference in serum free thiol levels compared to the implied reference group for categorical variables. Assumptions of normality of residual variance and homoscedasticity for linear regression analysis were fulfilled. Prior to further analysis, serum free thiol levels were ^2^log-transformed to facilitate results interpretation (expressed as per doubling). Logistic regression analyses were used to examine the association between serum free thiols and menopausal status. Survival distributions were created for tertiles of serum free thiol levels using Kaplan-Meier survival analysis and were compared with each other using log-rank tests. Survival time was defined from baseline (at time of serum sample withdrawal) until the date of the last visit participants attended, at time of an occurring cardiovascular (CV) event, death, or at January 1, 2010 (end of follow-up time). Cox proportional hazards regression analyses were used to assess the prospective association between serum free thiols and the risk of CV events. Results from Cox models were expressed as hazard ratios (HRs) with corresponding 95% confidence intervals (CIs). The proportionality of hazards assumption was checked for all predictor variables to confirm absence of violation. Multivariable Cox proportional hazards regression models were constructed in order to adjust for potential confounding factors. Stratified analyses of multivariable Cox regression models were performed to assess the associations between serum free thiols and the risk of CV events across various subgroups and to test for potential effect-modification through fitting models containing cross-product terms (*P*_interaction_). In addition, Cox regression models were performed using restricted cubic splines (RCS) with three knots to evaluate potential non-linearity of the associations between serum free thiols and the risk of CV events. Non-linearity was assessed by performing likelihood ratio tests, in which nested models were compared using linear or linear and cubic spline terms. Data analysis was performed using R version 3.5.2. (Vienna, Austria) and SPSS Statistics 25.0 software package (SPSS Inc., Chicago, IL, USA) and data visualization using GraphPad Prism version 8.0 (GraphPad Software, San Diego, CA, USA) and RStudio (version 1.2.1335, RStudio, Boston, MA). Two-tailed *P* ≤ 0.05 were considered statistically significant.

### Selection of Confounding Variables

**Figure 1:**
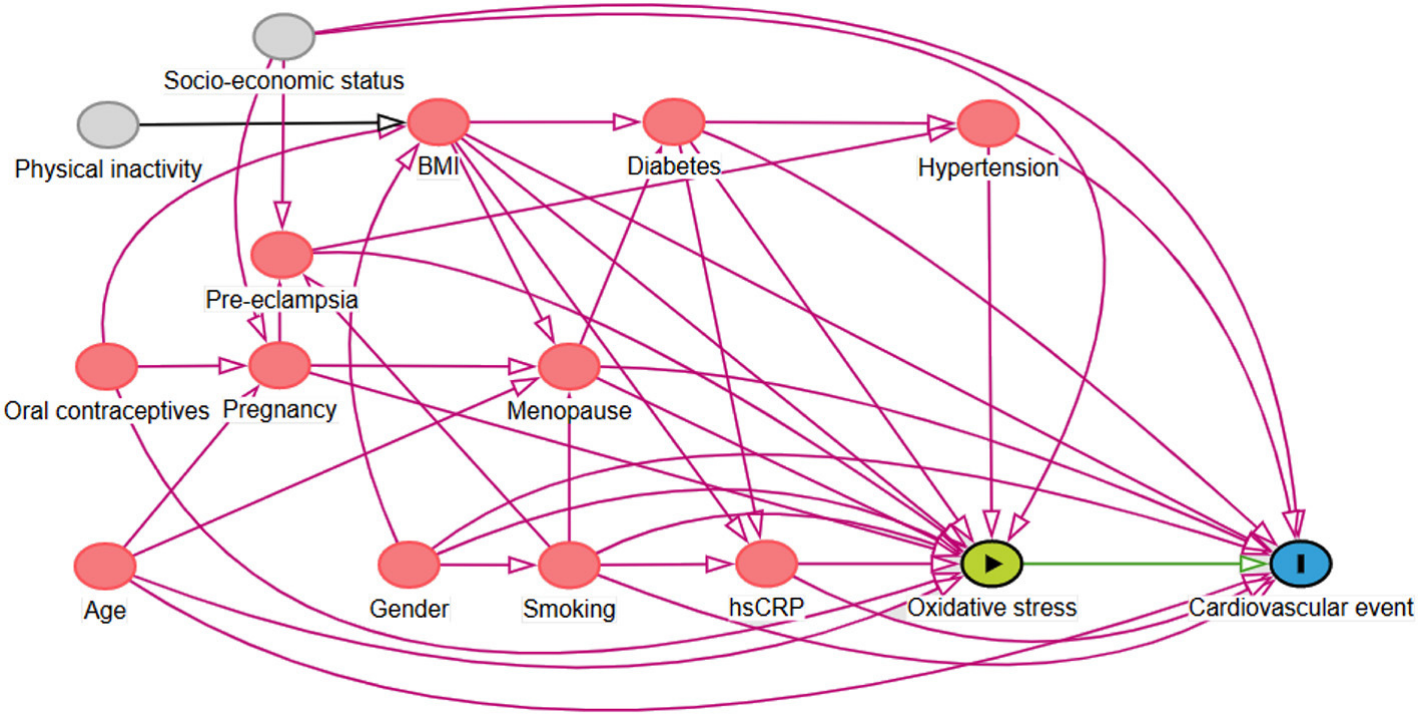
Directed Acyclic Graph (DAG) demonstrating the causal paths hypothesized to be underlying the relationship between serum free thiols, representing systemic oxidative stress, and the risk of cardiovascular (CV) events. Following from this graph, several confounders were accounted for in the Cox proportional hazards regression analysis.

In line with the guidelines for adjusting for confounders as described previously, we used causal models (directed acyclic graphs (DAGs) and their associated theory) to distinguish the appropriate set of confounders for estimating our effect of interest (21–23). Based on previously established theory in our field and respecting constraints imposed by time and logic, the DAG represents the causal mechanisms we hypothesize to be underlying the variables at hand (scenario 1, Figure 1) (24, 25). Arrows depict hypothesized causal (direct) effects between variables, whereas the absence of an arrow between two variables represents the assumption of no such direct effect. Focusing on the effect of oxidative stress on the outcome (cardiovascular events), we can now identify those variables for which conditioning in the analysis is necessary in order to obtain an unconfounded effect estimate in our statistical analysis. Consequently, we concluded that conditioning on menopausal status, body mass index (BMI), hypertension, diabetes, smoking, hs-CRP, and age as covariates in the analysis would provide an unconfounded effect estimate of oxidative stress on the outcome, the risk of cardiovascular events.

## Results

### Baseline Characteristics of the Study Population

For this study, a total of 2,980 women (1,469 premenopausal women and 1,511 postmenopausal women) were included in the analyses, of which serum free thiol levels and information about menopausal status were available. Distributions of serum free thiol levels across the total study population, as well as the specific pre- and postmenopausal groups, are shown in Supplementary Figure 1. A highly significant difference (*P* < 0.001) is shown between the latter two groups. The full study population was divided into tertiles according to levels of serum free thiols (*T1* < 4.60 μmol/g of protein; *T2* = 4.60–5.40 μmol/g of protein; *T3* > 5.40 μmol/g of protein). Mean concentration of serum free thiols in the full study population was 4.97 ± 1.01 μmol/g of protein.

Baseline study population characteristics (and other data) of the total group, as well as pre- and postmenopausal women specifically, are shown in Table 1, Supplementary Tables 1, 2, respectively, according to tertiles of serum free thiol concentrations (μmol/g). Across the total and both groups, significant (*P* < 0.001) differences were found for age, BMI, waist circumference, systolic blood pressure (SBP), hypertension, hs-CRP, creatinine, and eGFR. Furthermore, significant (*P* < 0.05) differences were observed across for alcohol use and AST.

**Table 1:** Baseline study population characteristics of women according to tertiles of serum free thiol concentrations (μmol/g).

**Variable**	**Total**	**T1** ** (<4.60)**	**T2** ** (4.60–5.40)**	**T3** ** (>5.40)**	***P*-value**
	***n*** **=** **2980**	***n*** **=** **993**	***n*** **=** **994**	***n*** **=** **993**	
Serum free thiols (μmol/g)	4.97 ± 1.01	3.88 ± 0.60	5.01 ± 0.22	6.05 ± 0.56	<0.001
Age (years)	50.9 [42.9; 59.5]	56.6 [46.6; 68.1]	50.7 [43.3; 58.3]	46.7 [40.7; 53.8]	<0.001
**Ethnicity**					
Caucasian, *n* (%)	2,841 (96.0)	954 (96.8)	944 (95.4)	943 (96.0)	
Asian, *n* (%)	57 (1.9)	16 (1.6)	21 (2.1)	20 (2.0)	0.620
Black, *n* (%)	31 (1.0)	8 (0.8)	15 (1.5)	8 (0.8)	
Other, *n* (%)	29 (1.0)	8 (0.8)	10 (1.0)	11 (1.1)	
BMI (kg/m^2^)	25.6 [23.0; 28.9]	26.9 [23.9; 30.3]	25.5 [23.2; 28.6]	24.6 [22.4; 27.5]	<0.001
Waist circumference (cm)	85 [78; 94]	89 [81; 98]	85 [78; 93]	82 [76; 90]	<0.001
**Smoking**					
Never, *n* (%)	989 (33.3)	349 (35.3)	325 (32.8)	315 (31.9)	
Former, *n* (%)	1,140 (38.4)	397 (40.1)	386 (39.0)	357 (36.1)	0.009
Current, *n* (%)	838 (28.2)	243 (24.6)	279 (28.2)	316 (32.0)	
**Alcohol use**					
No, *n* (%)	942 (31.6)	367 (37.0)	298 (30.0)	277 (27.9)	<0.001
Yes, *n* (%)	2,037 (68.4)	625 (63.0)	696 (70.0)	716 (72.1)	
**Blood pressure**					
SBP (mmHg)	117[108; 131]	124[111; 139]	116[107; 129]	114[106; 126]	<0.001
DBP (mmHg)	70 [64; 76]	71 [65; 77]	69 [64; 75]	69 [64; 75]	<0.001
**Co-morbidity**					
CVD history, *n* (%)	62 (2.1)	31 (3.1)	18 (1.8)	13 (1.3)	0.014
Hypertension, *n* (%)	763 (27.1)	374 (39.2)	224 (23.7)	165 (18.1)	<0.001
Diabetes, *n* (%)	66 (2.2)	34 (3.4)	19 (1.9)	13 (1.3)	0.004
**Laboratory parameters**					
Hemoglobin (mmol/L)	8.1 ± 0.6	8.1 ± 0.6	8.1 ± 0.6	8.0 ± 0.6	0.703
hs-CRP (mg/L)	1.4 [0.6; 3.2]	1.7 [0.8; 4.0]	1.4 [0.7; 3.2]	1.0 [0.5; 2.5]	<0.001
Albumin (g/L)	43.0 [42.0; 45.0]	43.0 [41.0; 45.0]	43.0 [42.0; 45.0]	43.0 [42.0; 45.0]	<0.001
Creatinine (μmol/L)	74.9 [68.8; 82.1]	77.0 [69.8; 85.2]	76.0 [69.8; 83.2]	72.9 [67.8; 80.1]	<0.001
eGFR (mL/min/1.73 m^2^)	92.7 ± 16.4	85.7 ± 17.2	93.4 ± 14.9	98.8 ± 14.3	<0.001
AST (U/L)	21.0 [18.0; 24.0]	21.0 [19.0; 25.0]	21.0 [18.0; 24.0]	20.0 [18.0; 23.0]	<0.001
ALT (U/L)	14.0 [11.0; 19.0]	15.0 [11.0; 20.0]	14.0 [11.0; 19.0]	14.0 [11.0; 19.0]	0.001
Total cholesterol (mmol/L)	5.5 ± 1.1	5.6 ± 1.0	5.5 ± 1.1	5.3 ± 1.1	<0.001
LDL-cholesterol (mmol/L)	3.3 [2.7; 4.1]	3.4 [2.8; 4.2]	3.2 [2.5; 3.9]	3.3 [2.5; 4.1]	0.262
HDL-cholesterol (mg/dL)	52.5 [45.3; 60.6]	51.6 [44.5; 59.6]	53.0 [45.3; 61.4]	52.8 [46.0; 61.0]	0.055
Triglycerides (mg/dL)	89.3 [65.6; 122.7]	96.1 [73.4; 127.3]	88.8 [64.7; 126.1]	81.8 [59.3; 112.5]	<0.001
Glucose (mmol/L)	4.7 [4.3; 5.1]	4.7 [4.4; 5.3]	4.7 [4.4; 5.1]	4.6 [4.3; 5.1]	<0.001
**Follow-up (10 years)**					
CV events, *n* (%)	112 (3.8)	53 (5.3)	36 (3.6)	23 (2.3)	0.002
Mortality, *n* (%)	94 (3.2)	51 (5.1)	22 (2.2)	21 (2.1)	<0.001
**Gynecological variables**					
**Menopausal status**					
Premenopausal, *n* (%)	1469 (49.3)	350 (35.2)	497 (50.0)	622 (62.6)	<0.001
Postmenopausal, *n* (%)	1511 (50.7)	643 (64.8)	497 (50.0)	371 (37.4)	
**Age at menopause**					
<37 years, *n* (%)	38 (2.6)	13 (2.1)	19 (4.0)	6 (1.7)	0.350
37–41 years, *n* (%)	122 (8.3)	55 (8.7)	36 (7.5)	31 (8.6)	
42–46 years, *n* (%)	307 (20.9)	139 (22.1)	93 (19.4)	75 (20.7)	
47–50 years, *n* (%)	469 (31.9)	187 (29.7)	156 (32.5)	126 (34.8)	
51–53 years, *n* (%)	367 (24.9)	156 (24.8)	127 (26.5)	84 (23.2)	
>53 years, *n* (%)	168 (11.4)	79 (12.6)	49 (10.2)	40 (11.0)	
**Pregnancy in past**					
No, *n* (%)	685 (23.1)	217 (22.0)	227 (22.9)	241 (24.4)	0.444
Yes, *n* (%)	2279 (76.9)	768 (78.0)	765 (77.1)	746 (75.6)	
**No. of children**					
0, *n* (%)	127 (5.4)	38 (4.8)	45 (5.7)	44 (5.7)	<0.001
1, *n* (%)	358 (12.0)	99 (12.5)	118 (15.1)	141 (18.2)	
2, *n* (%)	1159 (49.4)	350 (44.2)	402 (51.3)	407 (52.7)	
3, *n* (%)	497 (21.2)	201 (25.4)	152 (19.4)	144 (18.6)	
4, *n* (%)	120 (5.1)	56 (7.1)	44 (5.6)	20 (2.6)	
5, *n* (%)	47 (2.0)	22 (2.8)	12 (1.5)	13 (1.7)	
≥ 6, *n* (%)	40 (1.7)	25 (3.2)	11 (1.4)	4 (0.5)	
Hysterectomy, *n* (%)	70 (2.4)	20 (2.0)	25 (2.5)	25 (2.5)	0.693
Oophorectomy, *n* (%)	32 (1.1)	14 (1.4)	9 (0.9)	9 (0.9)	0.909
Current OCC use, *n* (%)	395 (13.3)	126 (12.7)	134 (13.5)	135 (13.6)	0.816
**Current female hormone use**					
Climacterium, *n* (%)	111 (3.8)	29 (2.9)	44 (4.5)	38 (3.8)	0.208
Other reasons, *n* (%)	77 (2.6)	20 (2.0)	23 (2.3)	34 (3.5)	0.113

*Data are presented as mean ± SD, median [IQR] in case of skewed variables or proportions n with corresponding percentages (%). Differences between tertiles of serum free thiol concentrations were tested using chi-square tests or Fisher's exact tests for nominal variables and one-way analysis of variance (ANOVA) in case of normally distributed continuous variables or Kruskal-Wallis tests in case of skewed continuous variables, as appropriate. P ≤ 0.05 were considered statistically significant. T1, tertile 1; T2, tertile 2; T3, tertile 3; BMI, body mass index; SBP, systolic blood pressure; DBP, diastolic blood pressure; CVD, cardiovascular disease; hs-CRP, high-sensitive C-reactive protein; eGFR, estimated glomerular filtration rate; AST, aspartate aminotransferase; ALT, alanine aminotransferase; LDL, low-density lipoprotein; HDL, high-density lipoprotein; OCC, oral contraceptives*.

In addition, specifically to premenopausal women, some significant differences between the tertiles that were found include current oral contraceptives (OCC) use (OCC were used more in lowest tertile, *P* < 0.001) and current female hormone use for reasons other than climacterium (*P* = 0.044). Specifically to postmenopausal women, some significant differences between the tertiles that were found include cardiovascular events (*P* = 0.032), mortality (*P* = 0.002), number of children (*P* = 0.004), hysterectomy (*P* = 0.022), and current female hormone use for climacterium (*P* = 0.049).

### Cross-Sectional Analyses

#### Associations Between Serum Free Thiols and Demographic, Clinical, Laboratory, and Gynecological Parameters

Univariable and multivariable linear regression analyses were performed to examine the relation between serum free thiol levels and menopausal status (Table 2, Supplementary Tables 3, 4, Supplementary Figures 2, 3). In the total cohort, variables that were significantly inversely associated with serum free thiols in multivariable analyses included age, waist circumference, and hs-CRP, whereas positive associations were observed for smoking status, eGFR, and triglycerides (Table 2).

**Table 2:** Univariable and multivariable linear regression analyses for identification of variables associating with serum free thiol levels in the study population.

**Variable**	**Univariable analysis**		**Multivariable analysis**	
	**St. Beta**	***P*-value**	**St. Beta**	***P*-value**
Age (years)	−0.297	<0.001	−0.132	<0.001
BMI (kg/m^2^)	−0.198	<0.001		
Waist circumference (cm)	−0.208	<0.001	−0.090	<0.001
Current smoking (%)	0.077	<0.001	0.066	0.001
Alcohol use (%)	0.087	<0.001		
Systolic blood pressure (mmHg)	−0.196	<0.001		
Diastolic blood pressure (mmHg)	−0.079	<0.001		
CVD history (%)	−0.062	0.001		
Hypertension (%)	−0.183	<0.001		
Diabetes (%)	−0.046	0.012		
**Laboratory parameters**				
Hemoglobin (mmol/L)	−0.003	0.873		
hs-CRP (mg/L)	−0.136	<0.001	−0.095	<0.001
Albumin (g/L)	0.050	0.007		
Creatinine (μmol/L)	−0.151	<0.001		
eGFR (mL/min/1.73 m^2^)	0.321	<0.001	0.219	<0.001
AST (U/L)	−0.081	<0.001		
ALT (U/L)	−0.030	0.105		
Total cholesterol (mmol/L)	−0.106	<0.001		
LDL-cholesterol (mmol/L)	−0.168	0.017		
HDL–cholesterol (mg/dL)	0.040	0.030		
Triglycerides (mg/dL)	−0.057	0.002	0.089	<0.001
Glucose (mmol/L)	−0.096	<0.001		
**Gynecological variables**				
Pregnancy in past (%)	−0.018	0.337		
No. of children (%)	−0.129	<0.001		
Hysterectomy (%)	0.017	0.356		
Oophorectomy (%)	−0.002	0.932		
Current OCC use (%)	0.005	0.782		
Current female hormone use (%)	0.020	0.288		

*BMI, body mass index; CVD, cardiovascular disease; hs-CRP, high-sensitive C-reactive protein; eGFR, estimated glomerular filtration rate; AST, aspartate aminotransferase; ALT, alanine aminotransferase; LDL, low-density lipoprotein; HDL, high-density lipoprotein; OCC, oral contraceptives*.

Among premenopausal women, significant inverse associations with serum free thiols were observed for SBP (*P* = 0.042, β = −0.056) and hs-CRP (*P* < 0.001, β = −0.148), whereas a positive association was observed for eGFR (*P* < 0.001, β = 0.167, Supplementary Figure 2). Of note, females with diabetes showed significantly lower levels of serum free thiols (Supplementary Figure 2D).

Among postmenopausal females, inverse associations were observed between serum free thiols and age and BMI, with patients of younger age and lower BMI having significantly higher serum free thiol levels (β = −0.312 and β = −0.181, respectively, both *P* < 0.001, Supplementary Figures 3A,B). Conversely, positive associations were observed for eGFR and hemoglobin levels (β = 0.301 and β = 0.052, respectively, both *P* < 0.001, Supplementary Figures 3C,D).

#### Associations Between Serum Free Thiols and Menopausal Status

To evaluate the association between menopausal status and serum free thiols, multivariable logistic regression analyses were performed (Table 3). Serum free thiols (^2^log-transformed) were significantly inversely associated with female menopausal status (OR 0.26 per doubling [0.20–0.33], *P* < 0.001, *Model 1*). After adjustment for all but one of our DAG-based confounders (BMI, SBP, diabetes, current smoking, and hs-CRP), this association remained statistically significant (OR 0.34 per doubling [0.25–0.46], *P* < 0.001, *Model 2*). After additional adjustment for a history of CVD, eGFR, and triglycerides, the association still remained significant (OR 0.70 per doubling [0.49–0.98], *P* = 0.039, *Model 3*). However, after adjusting for female age, this association shifted and lost statistical significance (OR 1.18 per doubling [0.69–2.00], *P* = 0.550, *Model 4*).

**Table 3:** Multivariable logistic regression analyses to investigate the association between serum free thiols (^2^log-transformed) and menopausal status.

	**Model 1**		**Model 2**		**Model 3**		**Model 4**	
	**OR [95% CI]**	***P*-value**	**OR [95% CI]**	***P*-value**	**OR [95% CI]**	***P*-value**	**OR [95% CI]**	***P*-value**
Serum free thiols (^2^log-transformed)	0.26 [0.20–0.33]	<0.001	0.34 [0.25–0.46]	<0.001	0.70 [0.49–0.98]	0.039	1.18 [0.69–2.00]	0.550
BMI			1.06 [1.04–1.09]	<0.001	1.03 [1.00–1.05]	0.055	1.04 [1.00–1.09]	0.041
SBP			1.04 [1.04–1.05]	<0.001	1.04 [1.03–1.04]	<0.001	0.99 [0.98–1.00]	0.021
Diabetes (reference = no)			2.08 [0.92–1.05]	0.080	1.85 [0.61–5.57]	0.276	0.79 [0.16–3.93]	0.773
Current smoking (reference = no)			1.10 [0.91–1.34]	0.322	1.00 [0.80–1.25]	0.990	1.69 [1.20–2.38]	0.002
hs-CRP			0.97 [0.95–0.99]	0.002	0.96 [0.93–0.98]	0.001	0.92 [0.89–0.96]	<0.001
History of CVD					1.51 [0.67–3.43]	0.323	1.04 [0.25–4.27]	0.962
Triglycerides					1.00 [1.00–1.01]	<0.001	1.00 [1.00–1.01]	0.191
eGFR					0.92 [0.92–0.93]	<0.001	0.99 [0.97–1.00]	0.042
Age							1.55 [1.48–1.61]	<0.001

*Model 1: crude; model 2, model 1, additionally adjusted for BMI, SBP, diabetes mellitus, current smoking and hs-CRP; model 3, model 2 additionally adjusted for history of CVD, triglycerides and eGFR; model 4, model 3, additionally adjusted for age. OR, odds ratio; BMI, body-mass index; SBP, systolic blood pressure; hs-CRP, high-sensitive C-reactive protein; CVD, cardiovascular diseases; eGFR, estimated glomerular filtration rate*.

### Prospective Analyses

#### Serum Free Thiols and the Risk of Cardiovascular (CV) Events

**Figure 2:**
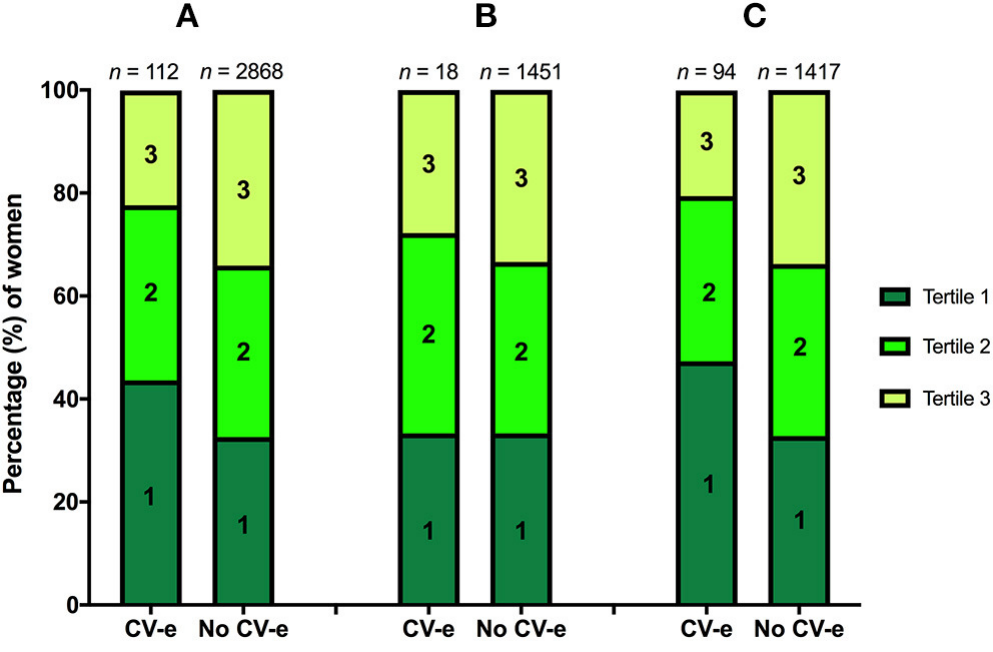
**(A–C)** Tertile distributions of serum free thiols stratified according to the occurrence of CV events (CV-e) during study follow-up among **(A)** the total cohort, **(B)** premenopausal women, and **(C)** postmenopausal women.

Tertile distributions of serum free thiols are shown in Figure 2, stratified according to the occurrence of CV events during 10 year study follow-up among (A) all study subjects (B) premenopausal women and (C) postmenopausal women. In the total cohort (*n* = 2,980), 112 females (3.8%) experienced CV events during the study follow-up period, of which the vast majority occurred in postmenopausal women (*n* = 94, 83.9%). Almost half of CV events among postmenopausal females (47.3%) occurred in females within the lowest tertile (<4.60 μmol/g) of serum free thiols at baseline, whereas approximately one-fifth (20.5%) occurred in the third tertile (>5.40 μmol/g) at baseline. Tertile distributions of premenopausal women were fairly similar (Figure 2B).

**Figure 3:**
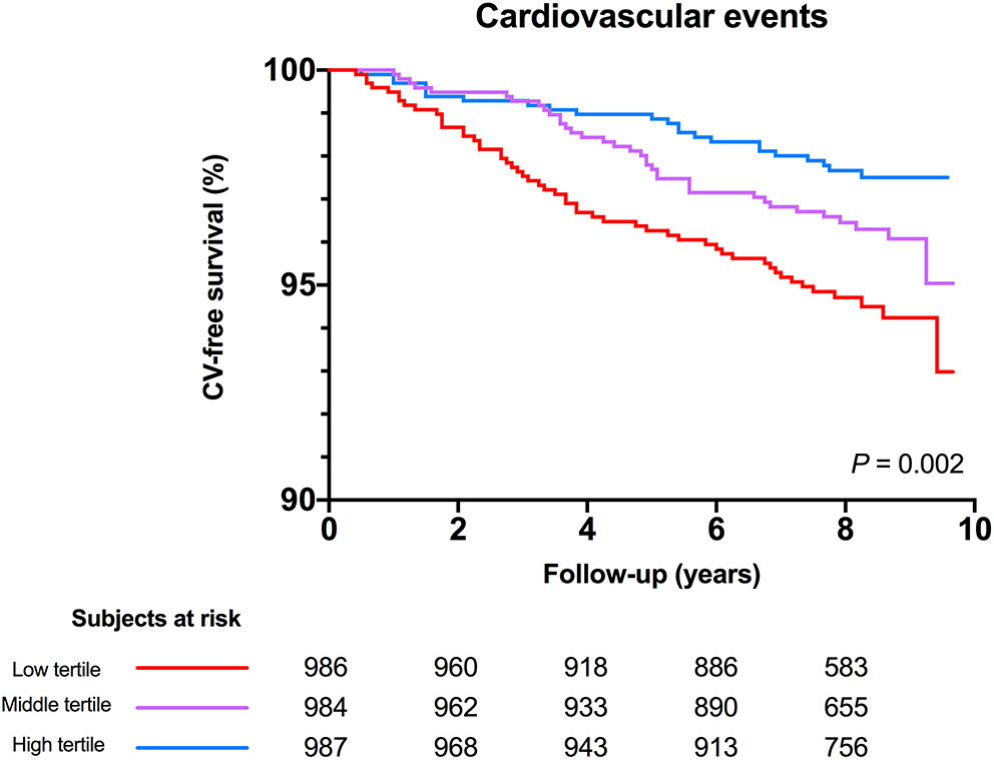
Kaplan-Meier survival distributions demonstrating CV-disease free survival among tertiles of serum free thiols. Highest rate of CV-events occurred in the lowest tertile of serum free thiols (log-rank test, *P* = 0.002).

Mean study follow-up was 7.9 ± 1.8 years, during which 112 (3.8%) CV events occurred. The highest rate of CV events occurred in the lowest tertile of serum free thiols (*n* = 53, 5.3%, *P* = 0.002). Kaplan-Meier survival analysis showed a statistically significant difference in survival distributions among tertiles of serum free thiols (log-rank test, *P* = 0.002, Figure 3).

**Figure 4:**
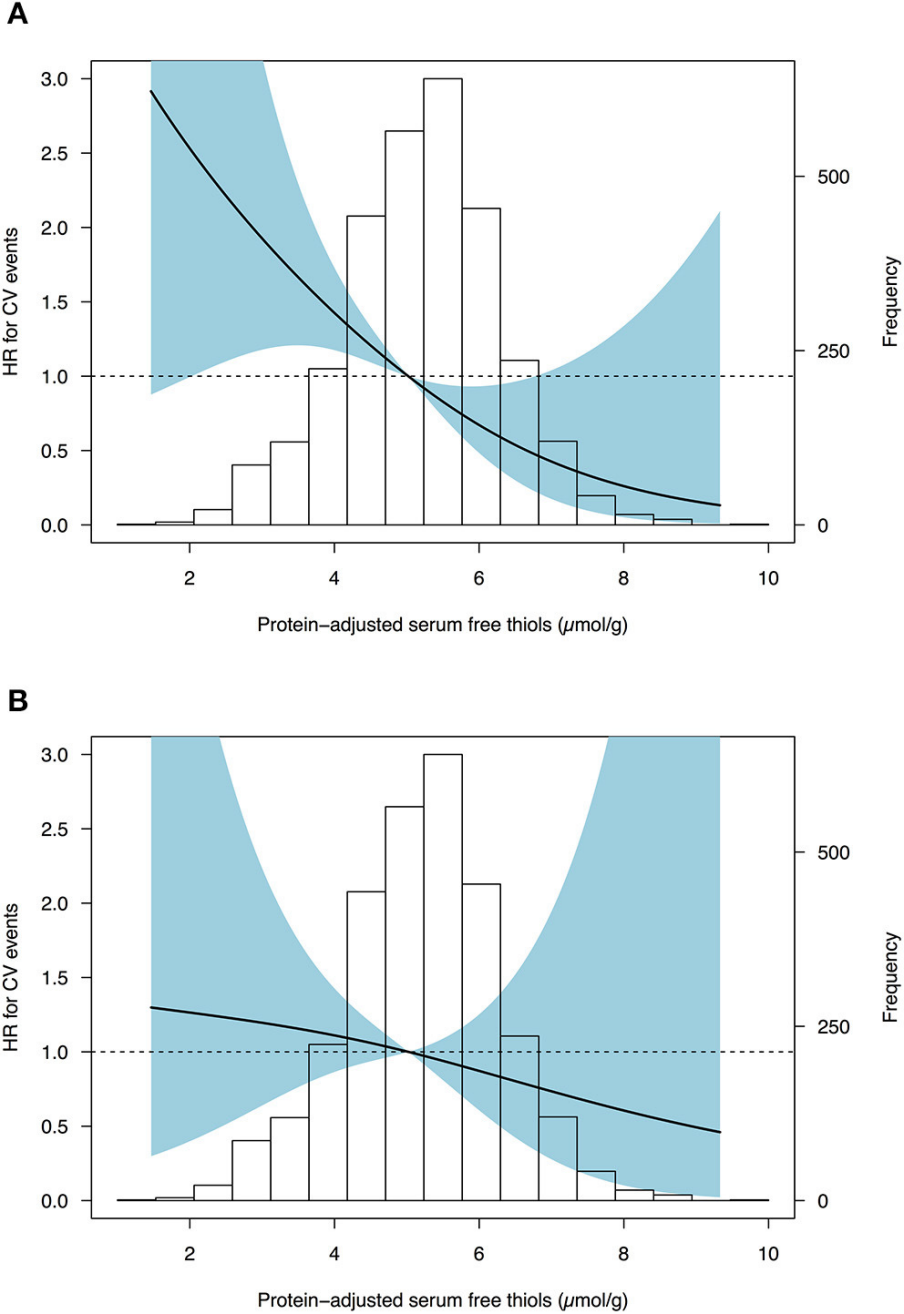
Restricted cubic splines (RCS) showing no deviance from linear associations of serum free thiols with the risk of CV events in females from the general population. **(A)** Cox proportional hazards regression analysis of serum free thiols with estimated associations with the risk of cardiovascular events based on restricted cubic splines with three knots (*Model 1*). **(B)** Cox proportional hazards regression analysis of serum free thiols with estimated associations derived from the fully adjusted model (*Model 4*). Median of serum free thiols was taken as reference standard (5.02 μmol/g). Likelihood ratio test for non-linearity was not statistically significant (χ^2^ = 0.14; *P* = 0.708). Light-blue shaded areas represent 95% confidence intervals.

Cox proportional hazard regression analyses showed a statistically significant association between serum free thiols and the risk of CV events (Table 4, *Model 1*, hazard ratio [HR] per doubling of serum free thiol concentrations 0.36 [0.22–0.61], *P* < 0.001). After adjustment for potential confounding factors (menopausal status, BMI, SBP, diabetes mellitus, current smoking, and hs-CRP) this association remained statistically significant (Table 4, *Model 3*, HR per doubling of serum free thiol concentrations 0.52 [0.27–0.97], *P* = 0.040). However, after additional adjustment for age, the association between serum free thiols and the risk of CV events lost its significance (Table 4, *Model 4*, HR per doubling 0.71 [0.36–1.40], *P* = 0.323). Restricted cubic splines showed no statistically significant deviation from linear association with the incidence of CV events for serum free thiols (Figure 4).

**Table 4:** Cox proportional hazards regression analyses of the association between ^2^log-transformed serum free thiols and the risk of CV events in the full female study population, with inclusion of potential confounding factors based on the directed acyclic graph (DAG).

**Serum free thiols and the risk of cardiovascular events**				
	**HR per doubling**	**Tertiles of serum free thiols**		
		<4.60 μmol/g	4.60–5.40 μmol/g	>5.40 μmol/g
Model 1	0.36 [0.22–0.61], *P* <0.001	1.00 (Reference)	0.67 [0.44–1.02], *P* = 0.062	0.42 [0.26–0.69], *P* = 0.001
Model 2	0.49 [0.29–0.86], *P* = 0.012	1.00 (Reference)	0.80 [0.52–1.23], *P* = 0.308	0.59 [0.36–0.97], *P* = 0.038
Model 3	0.52 [0.27–0.97], *P* = 0.040	1.00 (Reference)	0.84 [0.52–1.34], *P* = 0.457	0.61 [0.34–1.09], *P* = 0.096
Model 4	0.71 [0.36–1.40], *P* = 0.323	1.00 (Reference)	1.04 [0.62–1.70], *P* = 0.867	0.81 [0.44–1.49], *P* = 0.495

*Model 1: crude model; model 2: model 1, additionally adjusted for menopausal status; model 3: model 2, additionally adjusted for body-mass index (BMI), systolic blood pressure (SBP), diabetes mellitus, current smoking, high-sensitive C-reactive protein (hs-CRP); model 4 (based on DAG), model 3, additionally adjusted for age. Bold P-values indicate statistical significance. Abbreviations: HR, hazard ratio*.

#### Stratified Analyses for the Association Between Serum Free Thiols and the Risk of CV Events

Subsequently, the association between serum free thiols and the risk of CV events was assessed in various relevant subgroups (Table 5). Stratified analysis showed fairly consistent inverse associations between serum free thiols and the risk of CV events, with the exception of lower BMI (<25.0 kg/m^2^) and hypertension (though both non-significant). Stratification by a history of cardiovascular disease, alcohol consumption, and hypercholesterolemia resulted in significant interaction terms. Corresponding HRs were lower for females who had a history of CVD or those who consumed alcohol (both *P* < 0.001), whereas HRs were higher among females with hypercholesterolemia (*P* = 0.015).

**Table 5:** Stratified analyses for the association between ^2^log-transformed serum free thiols and the risk of cardiovascular events across various subgroups.

**Variable**	**Total**	**CV events**	**HR**	**95% CI**	***P*-value**
					**for interaction**
**Overall**	2,980	112	0.71	0.36–1.40	0.323
**Menopausal status**					
Premenopause	1,469	18	0.75	0.12–4.60	0.561
Postmenopause	1,511	94	0.66	0.31–1.40	
**BMI**					
<25.0 kg/m^2^	1,326	32	1.13	0.24–5.24	0.493
>25.0 kg/m^2^	1,646	80	0.63	0.29–1.36	
**Hypertension**					
No	2,049	37	0.72	0.23–2.25	0.702
Yes	763	69	0.88	0.34–2.25	
**History of CVD**					
No	2,918	99	0.88	0.42–1.86	<0.001
Yes	62	13	0.29	0.05–1.71	
**Diabetes**					
No	2,901	100	0.70	0.34–1.46	0.996
Yes	66	11	0.41	0.02–7.27	
**Current smoking**					
No	2,129	74	0.80	0.35–1.83	0.951
Yes	838	38	0.57	0.18–1.88	
**Alcohol consumption**					
No	942	62	0.77	0.31–1.91	<0.001
Yes	2,037	50	0.64	0.23–1.79	
**Hypercholesterolemia**					
No	2,012	51	0.57	0.20–1.58	0.015
Yes	801	55	1.08	0.40–2.90	

CV events, cardiovascular events; HR, hazard ratio; CI, confidence interval; BMI, body mass index; CVD, cardiovascular disease. ^*^Adjusted for potential confounding factors (menopausal status, BMI, SBP, diabetes mellitus, current smoking, hs-CRP).

## Discussion

The current study indicates significant associations between serum free thiols, as a marker for oxidative stress, and various markers for cardiovascular risk (i.e., age, BMI, hypertension). As expected, we also found a higher prevalence of cardiovascular events in postmenopausal women, as compared to premenopausal women. Furthermore, plotting out free thiol concentrations against menopausal status confirms that postmenopausal women have significantly lower concentrations of free thiols than premenopausal women, suggesting a relation between menopause and redox status. Moreover, Cox regression analyses revealed a significant association between free thiols and the risk of cardiovascular events after adjusting for potential confounding factors. However, after additional adjustment for age, this association lost its significance. Therefore, our data might suggest that age-related factors, other than menopausal status, play a more important role in the associations between oxidative stress and the occurrence of cardiovascular disease.

Our findings of lower levels of serum free thiols in postmenopausal women, representing relatively higher levels of systemic oxidative stress, are in line with studies describing how the decreased production of the antioxidant estrogen causes a pro-oxidant state in the female body (26–29). At higher concentrations, estrogen has a beneficial antioxidant effect by inhibiting the 8-hydroxylation of guanine DNA bases (27). In reproductive women, physiological serum concentrations of 17β-estradiol highly depend on the phase of the menstrual cycle. In general, levels range from 20 to 250 pg/mL during the early to midfollicular phase of the menstrual cycle, and peak between 30 and 650 pg/mL in the pre-ovulatory phase. Subsequently, serum 17β-estradiol decreases in the luteal phase to levels ranging from 20 to 300 pg/mL. In postmenopausal women, serum 17β-estradiol levels are substantially lower (<28 pg/mL) and are roughly comparable to levels measured in male humans. Previously, it has been shown that serum 17β-estradiol exhibits its antioxidant effects at physiological (1–10 nM) and slightly supraphysiological concentrations (0.01–1 μM for reproductive women (28). The antioxidant effect of estrogen has also been shown in animal studies, where the main production of estrogen was halted to observe the effects on oxidative stress, after which estrogen was administered to confirm the antioxidant findings (29). In humans, 17β-estradiol was found to stimulate expression and activity of manganese and extracellular superoxide dismutase (MnSOD and ecSOD), enzymes that break down ROS (30). Thereby, the decrease of this natural antioxidant hormone in postmenopausal women could increase their susceptibility to CVD. In line with this hypothesis, we showed a significant association between serum free thiols and cardiovascular disease. This confirms previously found associations between menopause and atherosclerosis (31). The exact mechanisms by which estrogen influences cardiovascular disease are not yet completely understood, but it is hypothesized that estrogen regulates the expression of specific cardiac genes (32). This has been shown in several animal studies; estrogen receptors are present on cardiomyocytes and play a role in expression of cardiac genes (33, 34). Furthermore, another human study suggested that maintaining a healthy antioxidant status contributes to protecting postmenopausal women from atherosclerotic CVD (35).

Oxidative stress is a common pathophysiological mechanism in many inflammatory and hypoxic conditions (7, 12). However, it is also part of the natural process of aging. In short, it results from overproduction of ROS in the presence of a weakening antioxidant system. This process preludes the susceptibility to diseases of aging, including diabetes, liver disease and atherosclerotic heart conditions. In women, this process is believed to be aggravated through the loss of production of female hormones, especially estrogen, although we could not fully confirm this hypothesis within the current study. Nonetheless, estrogen depletion further increases the levels of oxidative stress, causing cardiometabolic pathologies including heart disease and osteoporosis. At higher estrogen levels as measured in premenopausal women, there is a clear protective antioxidant effect of this hormone, while usually at lower concentrations estrogen appears to be pro-oxidant (36).

In the present study, we observed independent associations between serum free thiols and systolic blood pressure, hs-CRP and eGFR among premenopausal females, and with age, BMI, eGFR, and hemoglobin levels among postmenopausal females. These associations corroborate the findings of previous studies that examined serum free thiols in both healthy and diseased population cohorts (8, 12, 13, 37, 38). All these associations reflect a strong association between reduced systemic oxidative stress (i.e., lower levels of serum free thiols) and a favorable cardiovascular risk profile (e.g., lower age, BMI, blood pressure and hs-CRP, and higher eGFR and hemoglobin levels). For example, the inverse association between serum free thiols and hs-CRP is strongly consistent with these studies and confirms that systemic inflammation and oxidative stress are two highly associated pathophysiological phenomena. In addition, the inverse association with BMI corresponds with other studies demonstrating that higher levels of serum free thiols were associated with a more favorable cardiovascular risk profile (12, 13). In line with this, lower levels of serum free thiols have been associated with elevated parameters of visceral adiposity that may increase the risk of disease development (39). The observation that patients with type 2 diabetes mellitus have significantly lower levels of free thiols also confirms findings from previous studies (40, 41). Finally, the positive association between estimated kidney function and serum free thiols also validates previous work. In particular, a study that investigated serum free thiols in relation to cardiovascular risk parameters in renal transplant recipients suggested that free thiols could be used as a high-throughput screening tool for measuring whole-body redox status, as it was significantly associated with a better patient and graft survival in this study population (37).

Further, Cox proportional hazards regression analyses showed a significant association between free thiols and the risk of cardiovascular disease after correction for potential confounding factors (i.e., menopausal status, BMI, SBP, diabetes mellitus, current smoking, and hs-CRP). However, after additional adjustment for age, this association lost its significance. Therefore, other age-related factors might be more relevant in this association. In line with our findings, it was previously reported that aging has a remarkable effect on the cardiovascular system, leading to an increase in CVD (e.g., atherosclerosis, hypertension, myocardial infarction and stroke), independent from other traditional risk factors (8, 12, 36). Moreover, it is generally known that oxidative stress is positively associated with aging. Age-related changes in the thiol redox metabolome are reflected by an age-related increase in the production of reactive species, a decreased availability of antioxidants (such as estrogen), and a decreased ratio of reduced vs. oxidized forms of albumin (42). Similar to the present study, we previously reported that aging was associated with decreased systemic free thiol concentrations (7, 12, 39). However, it is still unknown what the exact age threshold is at which oxidative stress starts to play a prominent role. Larger confirmatory studies of longitudinal origin will be required to elucidate the aging trajectory of oxidative stress and the role of menopause in this process. Age not only remains a fundamental predictor of CVD, but future studies should focus on its exact role in the aging process.

The results of this study confirm what research has been increasingly showing for the past years; serum free thiols prove to be a reliable biomarker for oxidative stress, a major effector mechanism in many diseases and conditions (4, 8). Thiols are particularly interesting for research due to their susceptibility to therapeutic modulation, for example administering antioxidants (43). For instance, within the context of the present study, extracellular free thiols could theoretically be enhanced by hormone replacement therapy among postmenopausal women. In support of this hypothesis, our data revealed relatively more female hormone users among subjects within the highest tertile of serum free thiols, although limited information was available about the exact compounds or dosages that were used. One of the main strengths of our study is that it excels in the size of the study population. Most of the aforementioned studies that looked into the relationship between oxidative stress, CVD and menopause, averaged around a few hundred participants, whereas our study included several thousands. Additionally, to investigate the relationship with CVD, we included a follow-up of 10 years, whereas some other studies looked at it retrospectively or without follow-up. However, we do have to take into account potential limitations of our study. For example, the vast majority of our study population was of Caucasian ethnicity, causing uncertainty about whether our conclusions can be applied to all other ethnicities. In a lesser form, a similar uncertainty about the generalizability of our results should be reported regarding how the PREVEND study was only carried out in the northern part of the Netherlands. Additionally, the self-reported nature of some variables (e.g., menopausal status) could increase the risk of over/underestimation. Ideally, data on circulating sex hormone levels [i.e., estrogen, progesterone, testosterone, luteinizing hormone (LH), and follicle-stimulating hormone (FSH)] would have been used to strengthen the definition of menopausal status. Unfortunately, these data were lacking in the present cohort study and could therefore not be integrated. However, the subjective definition of menopausal status in our study is widely and internationally used in medical literature. Additionally, given the large sample size of the current study we deem it unlikely that this would significantly influence our results. Furthermore, it is unlikely that one single biomarker would be fully representative of the global extracellular redox state, as it could be skewed by the dynamic nature of oxidative stress as a pathophysiological entity. A combination of key components of the redox signaling network that represent integrative biomarkers would be preferable as they would combine read-outs of multiple redox-regulated metabolic pathways. However, such “redox metabolomics” approaches are still in their infancy as they are constrained by several (mainly methodological) issues (44). In addition, it is yet unclear what criteria potential redox biomarkers should fulfill in order to reliably assess the human redox system on a large-scale basis (4). In light of these considerations, the single quantification of serum free thiols is currently considered one of the most useful, high-throughput screening tools for measuring the whole-body redox status in translational settings.

In conclusion, serum free thiols were significantly reduced in postmenopausal women compared to women of reproductive age, even after adjustment for confounding factors, with exception of age. Similarly, we showed that serum free thiols, as a proxy of systemic oxidative stress, were significantly associated with the risk of cardiovascular events in the female general population, but significance vanished after age adjustment. Recapitulating, female (reproductive) aging is strongly associated with both oxidative stress and cardiovascular risk. More longitudinal studies are warranted to further disentangle the interplay between female menopause, human aging and the precise role of oxidative stress in both processes. In this respect, free thiols harbor considerable potential as translational redox biomarkers as they accurately represent the net state of extracellular oxidative stress. Future studies should however unravel the exact clinical utility of serum free thiol levels in the context of female cardiovascular risk management.

## Data Availability Statement

The datasets generated for this study are available on request to the corresponding author.

## Ethics Statement

The studies involving human participants were reviewed and approved by Medisch Ethische Toetsingscommissie, METc, University Medical Center Groningen. The patients/participants provided their written informed consent to participate in this study.

## Author Contributions

MB, AB, AA, RG, SB, SG, and HG were involved in conceptualization and study design. RG, SB, HG, and AP were responsible for funding acquisition and resources. LK, RG, SB, AP, and HG collected all study data. MB, AB, AA, LK, and SB-v performed data curation, data analysis, and visualization. MB, AB, AA, SG, and HG wrote the first draft of the manuscript. All authors contributed to results interpretation, critically reviewed the manuscript, contributed to manuscript revision, and read and approved the final version of the manuscript.

## Conflict of Interest

The authors declare that the research was conducted in the absence of any commercial or financial relationships that could be construed as a potential conflict of interest.

## Supplementary Material

Supplementary Figure 1 Serum free thiols are significantly reduced in postmenopausal women (^***^*P* < 0.001).

Supplementary Figure 2 **(A–D)** Serum free thiols show significant associations with **(A)** systolic blood pressure, **(B)** high-sensitive C-reactive protein (hs-CRP), and **(C)** estimated glomerular filtration rate (eGFR) in premenopausal females. **(D)** Premenopausal females having diabetes mellitus show significantly lower levels of serum free thiols (^***^*P* < 0.001).

Supplementary Figure 3 **(A–D)** In postmenopausal females, serum free thiols showed significant inverse associations with **(A)** age and **(B)** body mass index (BMI), whereas positive associations were observed for **(C)** estimated glomerular filtration rate (eGFR) and **(D)** hemoglobin levels.
