# Supplementary figures and images for: Systemic Oxidative Stress, Aging and the Risk of Cardiovascular Events in the General Female Population
Source: Front Cardiovasc Med. 2021 Feb 9;8:630543. doi: 10.3389/fcvm.2021.630543 (PMC7900172; doi:10.3389/fcvm.2021.630543)

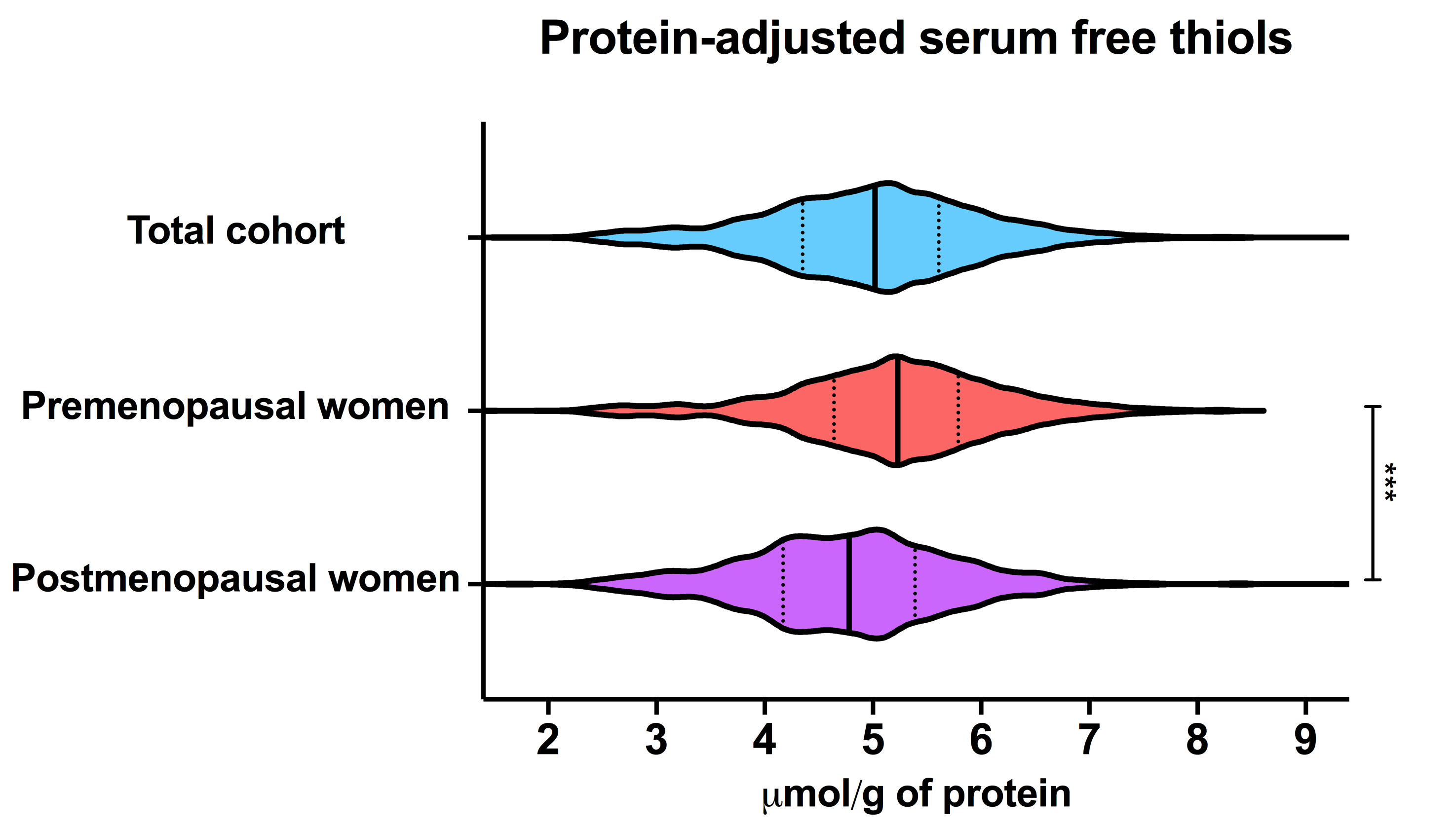

Supplement: Supplementary Figure 1 — Serum free thiols are significantly reduced in postmenopausal women (***P < 0.001). [file Image_1.TIFF]

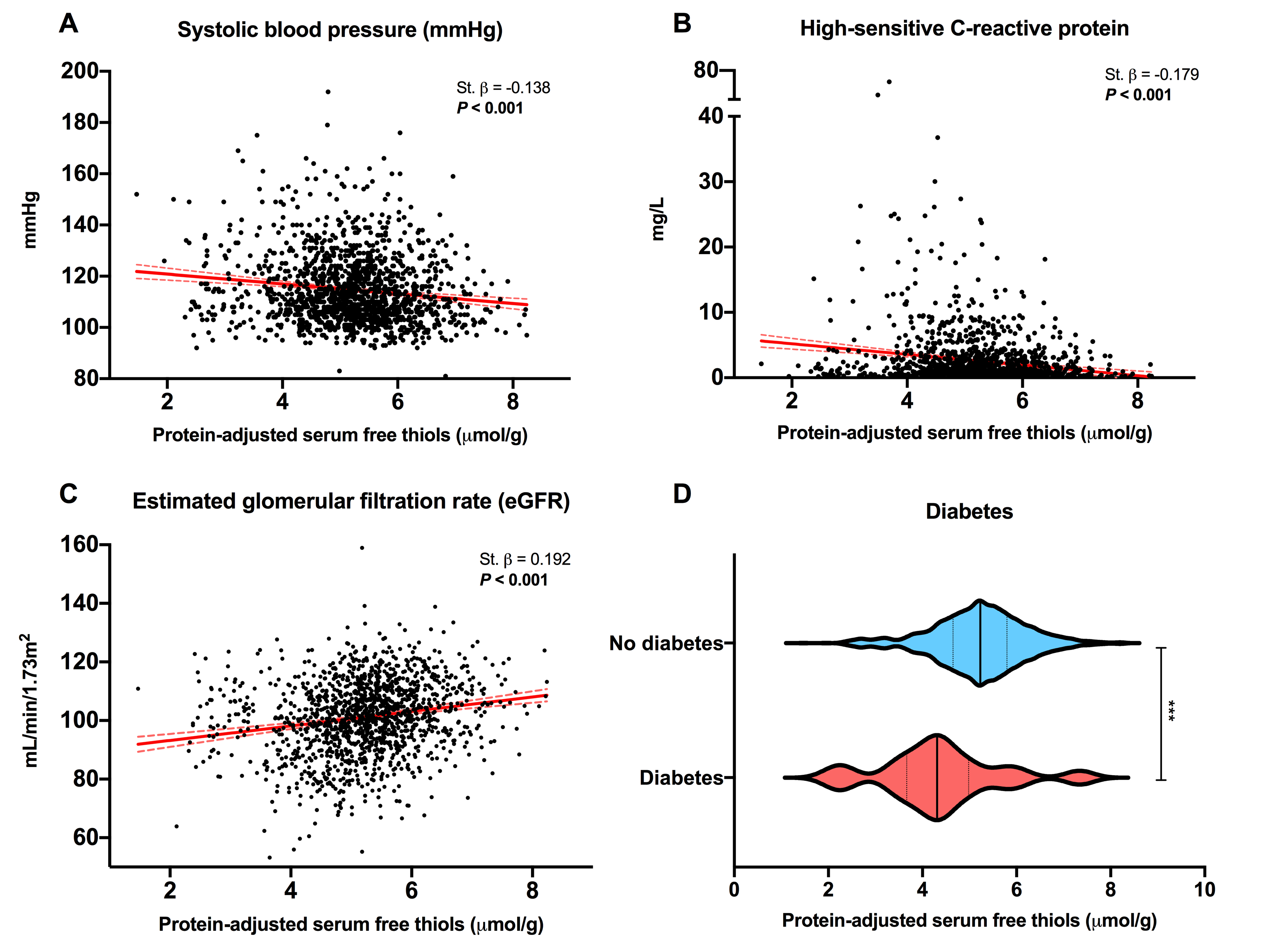

Supplement: Supplementary Figure 2 — (A–D) Serum free thiols show significant associations with (A) systolic blood pressure, (B) high-sensitive C-reactive protein (hs-CRP), and (C) estimated glomerular filtration rate (eGFR) in premenopausal females. (D) Premenopausal females having diabetes mellitus show significantly lower levels of serum free thiols (***P < 0.001). [file Image_2.TIFF]

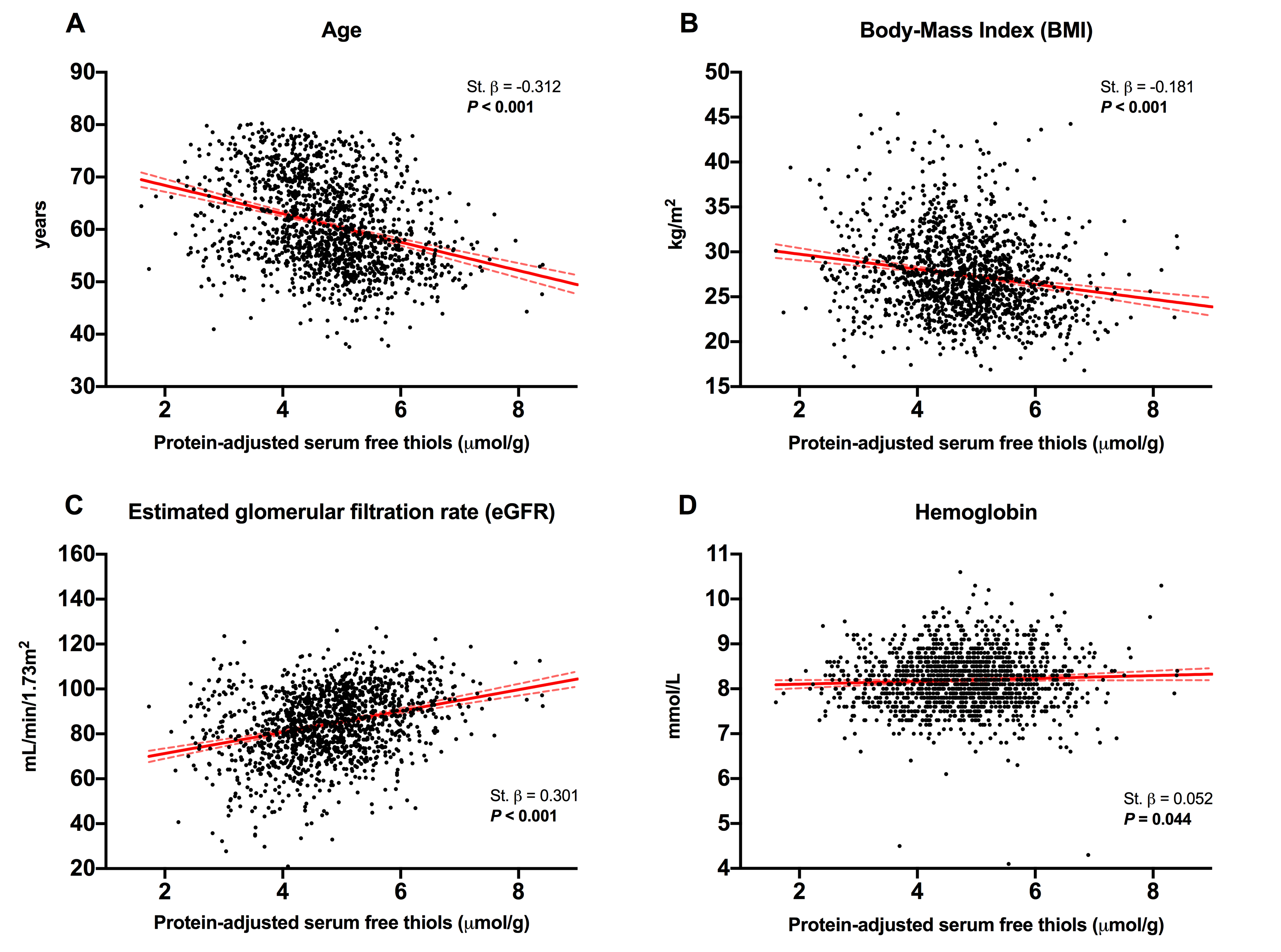

Supplement: Supplementary Figure 3 — (A–D) In postmenopausal females, serum free thiols showed significant inverse associations with (A) age and (B) body mass index (BMI), whereas positive associations were observed for (C) estimated glomerular filtration rate (eGFR) and (D) hemoglobin levels. [file Image_3.TIFF]
