# Supplementary material for: Systemic Oxidative Stress, Aging and the Risk of Cardiovascular Events in the General Female Population
Source: Front Cardiovasc Med. 2021 Feb 9;8:630543. doi: 10.3389/fcvm.2021.630543 (PMC7900172; doi:10.3389/fcvm.2021.630543)
Supplement: Supplementary file 4 [file Data_Sheet_1.docx]

**Supplementary Material**

**Supplementary Table S1**. Baseline study population characteristics of premenopausal women according to tertiles of serum free thiol concentrations (μmol/g).

| **Variable** | **Total** | **T1**  **(< 4.87)** | **T2**  **(4.87-5.59)** | **T3**  **(> 5.59)** | ***P*-value^*^** |
| --- | --- | --- | --- | --- | --- |
|  | *n* = 1469 | *n* = 489 | *n* = 490 | *n* = 490 |  |
| Serum free thiols (μmol/g) | 5.2 ± 1.0 | 4.1 ± 0.7 | 5.2 ± 0.2 | 6.2 ± 0.5 | <0.001 |
| Age (years) | 43.0 [38.8;47.1] | 43.4 [39.1;48.2] | 43.3 [38.9;47.2] | 42.1 [38.3;46.3] | 0.011 |
| Ethnicity  Caucasian, *n* (%)  Asian, *n* (%)  Black, *n* (%)  Other, *n* (%) | 1374 (94.4)  38 (2.6)  24 (1.6)  20 (1.4) | 459 (94.4)  10 (2.1)  8 (1.6)  9 (1.9) | 456 (93.4)  14 (2.9)  11 (2.3)  7 (1.4) | 459 (95.2)  14 (2.9)  5 (1.0)  4 (0.8) | 0.547 |
| BMI (kg/m^2^) | 24.4 [22.3;27.4] | 24.8 [22.6;28.4] | 24.4 [22.6;27.2] | 23.8 [21.9;26.5] | <0.001 |
| Waist circumference (cm) | 82 [76;89] | 83 [76;92] | 82 [76;89] | 80 [75;87] | <0.001 |
| Smoking  Never, *n* (%)  Former, *n* (%)  Current, *n* (%) | 488 (33.2)  530 (36.1)  443 (30.3) | 174 (35.6)  172 (35.2)  140 (28.6) | 166 (33.9)  185 (37.8)  136 (27.8) | 148 (30.2)  173 (35.3)  167 (34.1) | 0.585 |
| Alcohol use  No, *n* (%)  Yes, *n* (%) | 404 (27.5)  1065 (72.5) | 151 (30.9)  338 (69.1) | 120 (24.5)  370 (75.5) | 133 (27.1)  357 (72.9) | 0.080 |
| Blood pressure  SBP (mmHg)  DBP (mmHg) | 112[105;122]  68 [63;73] | 113[106;124]  68 [63;74] | 112[106;122]  68 [63;74] | 110[103;119]  67 [62;72] | <0.001  0.002 |
| Co-morbidity |  |  |  |  |  |
| CVD history, *n* (%)  Hypertension, *n* (%)  Diabetes, n (%) | 17 (1.2)  158 (11.4)  17 (1.2) | 8 (1.6)  76 (16.2)  12 (2.5) | 6 (1.2)  41 (8.7)  2 (0.4) | 3 (0.6)  41 (9.1)  3 (0.6) | 0.321  < 0.001  0.004 |
| Laboratory parameters |  |  |  |  |  |
| Hemoglobin (mmol/L) | 7.9 ± 0.6 | 7.9 ± 0.7 | 7.9 ± 0.6 | 8.0 ± 0.6 | 0.383 |
| hs-CRP (mg/L) | 1.1 [0.5;2.9] | 1.4 [0.6;4.0] | 1.1 [0.5;2.9] | 0.9 [0.4;2.1] | <0.001 |
| Albumin (g/L) | 43.0 [42.0;45.0] | 43.0 [41.0;45.0] | 43.0 [42.0;45.0] | 43.0 [42.0;45.0] | 0.075 |
| Creatinine (μmol/L) | 74.9 [68.8;81.1] | 76.0 [69.8;83.7] | 74.9 [68.8;81.1] | 71.9 [67.8;79.1] | <0.001 |
| eGFR (mL/min/1.73m^2^) | 101.2 ± 12.6 | 97.6 ± 13.1 | 101.7 ± 12.3 | 104.1 ± 11.5 | <0.001 |
| AST (U/L) | 19.0 [17.0;22.0] | 20.0 [17.0;23.0] | 19.0 [17.0;22.0] | 19.0 [17.0;22.0] | 0.032 |
| ALT (U/L) | 13.0 [10.0;17.0] | 13.0 [11.0;17.5] | 13.0 [10.0;17.0] | 13.0 [10.0;17.0] | 0.132 |
| Total cholesterol (mmol/L) | 4.9 [4.4;5.5] | 5.0 [4.4;5.6] | 5.0 [4.4;5.6] | 4.8 [4.3;5.4] | 0.004 |
| LDL-cholesterol (mmol/L) | 3.2 [2.7;3.8] | 3.3 [2.8;3.9] | 2.9 [2.3;3.7] | 3.1 [2.6;3.7] | 0.214 |
| HDL-cholesterol (mg/dL) | 53.5 ± 11.2 | 53.3 ± 11.1 | 53.5 ± 11.9 | 53.6 ± 10.6 | 0.891 |
| Triglycerides (mg/dL) | 77.8 [58.3;106.9] | 81.9 [61.4;114.4] | 78.0 [58.5;107.8] | 72.0 [55.3;98.2] | <0.001 |
| Glucose (mmol/L) | 4.5 [4.3;5.0] | 4.5 [4.2;5.0] | 4.6 [4.3;5.0] | 4.5 [4.3;4.8] | 0.594 |
| Follow-up (10 years) |  |  |  |  |  |
| CV events, *n* (%) | 18 (1.2) | 6 (1.2) | 7 (1.4) | 5 (1.0) | 0.845 |
| Mortality, *n* (%) | 17 (1.2) | 3 (0.6) | 4 (0.8) | 10 (2.0) | 0.078 |
| Gynecological variables |  |  |  |  |  |
| Pregnancy in past  No, *n* (%)  Yes, *n* (%) | 445 (30.4)  1018 (69.6) | 167 (34.2)  322 (65.8) | 136 (27.9)  352 (72.1) | 142 (29.2)  344 (70.8) | 0.080 |
| No. of children  0, *n* (%)  1, *n* (%)  2, *n* (%)  3, *n* (%)  4, *n* (%)  5, *n* (%)  ≥ 6, *n* (%) | 82 (7.8)  202 (19.1)  542 (51.4)  184 (17.4)  30 (2.8)  9 (0.9)  6 (0.6) | 31 (9.3)  62 (18.5)  156 (46.6)  67 (20.0)  12 (3.6)  4 (1.2)  3 (0.9) | 23 (6.4)  64 (17.8)  201 (56.0)  56 (15.6)  11 (3.1)  1 (0.3)  3 (0.8) | 28 (7.8)  76 (21.1)  185 (51.2)  61 (16.9)  7 (1.9)  4 (1.1)  0 | 0.247 |
| Hysterectomy, *n* (%) | 2 (0.1) | 0 | 2 (0.4) | 0 | 0.135 |
| Oophorectomy, *n* (%) | 3 (0.2) | 1 (0.2) | 2 (0.4) | 0 | 0.238 |
| Current OCC use, *n* (%) | 370 (25.2) | 154 (31.6) | 124 (25.3) | 92 (18.8) | < 0.001 |
| Current female hormone use  Climacterium, *n* (%)  Other reasons, *n* (%) | 29 (2.0)  32 (2.2) | 10 (2.0)  9 (1.8) | 8 (1.6)  6 (1.2) | 11 (2.2)  17 (3.5) | 0.784  0.044 |

Data are presented as mean ± SD, median [IQR] in case of skewed variables or proportions n with corresponding percentages (%). Differences between tertiles of serum free thiol concentrations were tested using chi-square tests or Fisher’s exact tests for nominal variables and one-way analysis of variance (ANOVA) in case of normally distributed continuous variables or Kruskal-Wallis tests in case of skewed continuous variables, as appropriate. Bold P-values indicate statistical significance. P-values ≤ 0.05 were considered statistically significant. Abbreviations: T1, tertile 1; T2, tertile 2; T3, tertile 3; BMI, body mass index; SBP, systolic blood pressure; DBP, diastolic blood pressure; CVD, cardiovascular disease; hs-CRP, high-sensitive C-reactive protein; eGFR, estimated glomerular filtration rate; AST, asparate aminotransferase; ALT, alanine aminotransferase; LDL, low-density lipoprotein; HDL, high-density lipoprotein; OCC, oral contraceptives.

**Supplementary Table S2**. Baseline study population characteristics of postmenopausal women according to tertiles of serum free thiol concentrations (μmol/g).

| **Variable** | **Total** | **T1**  **(< 4.36)** | **T2**  **(4.36-5.16)** | **T3**  **(> 5.16)** | ***P*-value^*^** |
| --- | --- | --- | --- | --- | --- |
|  | *n* = 1511 | *n* = 503 | *n* = 504 | *n* = 504 |  |
| Serum free thiols (μmol/g) | 4.8 ± 1.0 | 3.7 ± 0.5 | 4.8 ± 0.2 | 5.8 ± 0.6 | <0.001 |
| Age (years) | 59.4 [54.5;67.7] | 65.3 [57.5;71.3] | 59.1 [54.4;66.1] | 56.3 [52.4;62.7] | <0.001 |
| Ethnicity  Caucasian, *n* (%)  Asian, *n* (%)  Black, *n* (%)  Other, *n* (%) | 1467 (97.7)  19 (1.3)  7 (0.5)  9 (0.6) | 489 (98.0)  6 (1.2)  4 (0.8)  0 | 488 (97.2)  9 (1.8)  1 (0.2)  4 (0.8) | 490 (97.8)  4 (0.8)  2 (0.4)  5 (1.0) | 0.193 |
| BMI (kg/m^2^) | 26.9 [24.2;30.0] | 27.8 [25.0;31.2] | 26.6 [24.2;29.6] | 26.0 [23.6;29.1] | <0.001 |
| Waist circumference (cm) | 89.0 [81.0;98.0] | 91.0 [83.0;101.0] | 88.0 [81.0;96.0] | 87.0 [80.0;96.0] | <0.001 |
| Smoking  Never, *n* (%)  Former, *n* (%)  Current, *n* (%) | 501 (33.2)  610 (40.4)  395 (26.1) | 182 (36.2)  222 (44.1)  98 (19.5) | 161 (31.9)  194 (38.5)  147 (29.2) | 158 (31.3)  194 (38.5)  150 (29.8) | 0.006 |
| Alcohol use  No, *n* (%)  Yes, *n* (%) | 538 (35.6)  972 (64.4) | 207 (41.2)  295 (58.8) | 175 (34.7)  329 (65.3) | 156 (31.0)  348 (69.0) | 0.003 |
| Blood pressure  SBP (mmHg)  DBP (mmHg) | 125 [112;140]  71[66;78] | 130 [117;144]  72[66;78] | 123 [111;138]  71[66;77] | 121 [110;138]  72[67;78] | <0.001  0.412 |
| Co-morbidity |  |  |  |  |  |
| CVD history, *n* (%)  Hypertension, *n* (%)  Diabetes, n (%) | 45 (3.0)  605 (42.5)  49 (3.2) | 22 (4.4)  251 (52.0)  16 (3.2) | 12 (2.4)  181 (37.8)  16 (3.2) | 11 (2.2)  173 (37.5)  17 (3.4) | 0.077  < 0.001  0.982 |
| Laboratory parameters |  |  |  |  |  |
| Hemoglobin (mmol/L) | 8.2 ± 0.6 | 8.1 ± 0.6 | 8.2 ± 0.6 | 8.2 ± 0.7 | 0.132 |
| hs-CRP (mg/L) | 1.6 [0.8;3.4] | 2.0 [1.0;4.1] | 1.5 [0.8;3.3] | 1.5 [0.7;3.1] | <0.001 |
| Albumin (g/L) | 43.4 ± 3.1 | 42.9 ± 2.6 | 43.6 ± 3.9 | 43.7 ± 2.7 | <0.001 |
| Creatinine (μmol/L) | 76.0 [69.8;84.2] | 78.0 [70.8;86.2] | 77.0 [69.8;83.2] | 73.9 [67.8;81.1] | <0.001 |
| eGFR (mL/min/1.73m^2^) | 85.7 [74.7;95.7] | 79.4 [68.3;89.0] | 85.8 [76.3;95.0] | 91.0 [80.7;100.2] | <0.001 |
| AST (U/L) | 22.0 [19.0;26.0] | 23.0 [20.0;26.0] | 22.0 [19.0;25.0] | 22.0 [19.0;25.0] | 0.002 |
| ALT (U/L) | 16.0 [12.0;21.0] | 16.0 [12.0;21.0] | 16.0 [12.0;21.0] | 16.0 [12.0;22.0] | 0.778 |
| Total cholesterol (mmol/L) | 5.9 ± 1.0 | 5.8 ± 1.0 | 5.9 ± 1.0 | 5.9 ± 1.1 | 0.553 |
| LDL-cholesterol (mmol/L) | 3.5 [2.7;4.3] | 3.7 [3.1;4.6] | 3.2 [2.5;4.0] | 3.5 [2.6;4.5] | 0.053 |
| HDL-cholesterol (mg/dL) | 52.5 [45.1;61.0] | 50.9 [44.4;59.0] | 53.9 [46.0;62.2] | 52.6 [45.3;61.8] | 0.026 |
| Triglycerides (mg/dL) | 101.7 [75.8;139.1] | 103.8 [78.0;134.7] | 101.1 [75.4;140.5] | 100.0 [72.0;144.0] | 0.534 |
| Glucose (mmol/L) | 4.8 [4.5;5.3] | 4.8 [4.5;5.4] | 4.8 [4.5;5.3] | 4.7 [4.4;5.3] | 0.154 |
| Follow-up (10 years) |  |  |  |  |  |
| CV events, *n* (%) | 94 (6.2) | 41 (8.2) | 32 (6.3) | 21 (4.2) | 0.032 |
| Mortality, *n* (%) | 77 (5.1) | 39 (7.8) | 23 (4.6) | 15 (3.0) | 0.002 |
| Gynecological variables |  |  |  |  |  |
| Age at menopause  < 37 years, *n* (%)  37 - 41 years, *n* (%)  42 – 46 years, *n* (%)  47 – 50 years, *n* (%)  51 – 53 years, *n* (%)  > 53 years, *n* (%) | 37 (2.6)  118 (8.2)  302 (21.1)  454 (31.7)  358 (25.)  164 (11.4) | 8 (1.6)  45 (9.3)  109 (22.5)  143 (29.5)  114 (23.5)  66 (13.6) | 15 (3.2)  33 (7.0)  94 (20.0)  151 (32.1)  132 (28.0)  46 (9.8) | 14 (2.9)  40 (8.4)  99 (20.8)  160 (33.5)  112 (23.5)  52 (10.9) | 0.289 |
| Pregnancy in past  No, *n* (%)  Yes, *n* (%) | 240 (16.0)  1261 (84.0) | 82 (16.5)  414 (83.5) | 79 (15.7)  423 (84.3) | 79 (16.0)  424 (84.3) | 0.922 |
| No. of children  0, *n* (%)  1, *n* (%)  2, *n* (%)  3, *n* (%)  4, *n* (%)  5, *n* (%)  ≥ 6, *n* (%) | 45 (3.5)  156 (12.1)  617 (47.7)  313 (24.2)  90 (7.0)  38 (2.9)  34 (2.6) | 13 (3.0)  46 (10.7)  174 (40.7)  119 (27.8)  44 (10.3)  15 (3.5)  17 (4.0) | 17 (3.9)  54 (12.5)  213 (49.4)  98 (22.7)  26 (6.0)  12 (2.8)  11 (2.6) | 15 (3.5)  56 (12.9)  230 (53.0)  96 (22.1)  20 (4.6)  11 (2.5)  6 (1.4) | 0.004 |
| Hysterectomy, *n* (%) | 68 (4.5) | 16 (3.2) | 19 (3.8) | 33 (6.6) | 0.022 |
| Oophorectomy, *n* (%) | 29 (2.0) | 12 (2.4) | 5 (1.0) | 12 (2.4) | 0.239 |
| Current OCC use, *n* (%) | 25 (1.7) | 5 (1.0) | 12 (2.4) | 8 (1.6) | 0.224 |
| Current female hormone use  Climacterium, *n* (%)  Other reasons, *n* (%) | 82 (5.5)  45 (3.0) | 17 (3.4)  12 (2.4) | 32 (6.4)  12 (2.4) | 33 (6.6)  21 (4.3) | 0.049  0.150 |

Data are presented as mean ± SD, median [IQR] in case of skewed variables or proportions n with corresponding percentages (%). Differences between tertiles of serum free thiol concentrations were tested using chi-square tests or Fisher’s exact tests for nominal variables and one-way analysis of variance (ANOVA) in case of normally distributed continuous variables or Kruskal-Wallis tests in case of skewed continuous variables, as appropriate. Bold P-values indicate statistical significance. P-values ≤ 0.05 were considered statistically significant. Abbreviations: T1, tertile 1; T2, tertile 2; T3, tertile 3; BMI, body mass index; SBP, systolic blood pressure; DBP, diastolic blood pressure; CVD, cardiovascular disease; hs-CRP, high-sensitive C-reactive protein; eGFR, estimated glomerular filtration rate; AST, aspartate aminotransferase; ALT, alanine aminotransferase; LDL, low-density lipoprotein; HDL, high-density lipoprotein; OCC, oral contraceptives.

**Supplementary Table S3**. Univariable and multivariable linear regression analyses for identification of variables associating with serum free thiol levels in premenopausal women.

| **Variable** | **Univariable analysis** | | **Multivariable analysis** | |
| --- | --- | --- | --- | --- |
|  | St. Beta | *P*-value | St. Beta | *P*-value |
| Age (years) | -0.074 | 0.005 |  |  |
| BMI (kg/m^2^) | -0.139 | <0.001 |  |  |
| Waist circumference (cm) | -0.142 | <0.001 |  |  |
| Current smoking (%) | 0.032 | 0.219 |  |  |
| Alcohol use (%) | 0.039 | 0.135 |  |  |
| Systolic blood pressure (mmHg) | -0.138 | <0.001 | -0.056 | 0.042 |
| Diastolic blood pressure (mmHg) | -0.079 | 0.002 |  |  |
| CVD history (%) | -0.032 | 0.224 |  |  |
| Hypertension (%) | -0.105 | <0.001 |  |  |
| Diabetes (%) | -0.090 | 0.001 | -0.100 | <0.001 |
| Laboratory parameters |  |  |  |  |
| Hemoglobin (mmol/L) | 0.037 | 0.153 |  |  |
| hs-CRP (mg/L) | -0.179 | <0.001 | -0.148 | <0.001 |
| Albumin (g/L) | 0.018 | 0.483 |  |  |
| Creatinine (μmol/L) | -0.142 | <0.001 |  |  |
| eGFR (mL/min/1.73m^2^) | 0.192 | <0.001 | 0.167 | <0.001 |
| AST (U/L) | -0.013 | 0.628 |  |  |
| ALT (U/L) | 0.010 | 0.690 |  |  |
| Total cholesterol (mmol/L) | -0.070 | 0.008 |  |  |
| LDL-cholesterol (mmol/L) | -0.270 | 0.010 |  |  |
| HDL-cholesterol (mg/dL) | 0.023 | 0.381 |  |  |
| Triglycerides (mg/dL) | -0.096 | <0.001 |  |  |
| Glucose (mmol/L) | -0.076 | 0.004 |  |  |
| Gynecological variables |  |  |  |  |
| Pregnancy in past (%) | 0.023 | 0.375 |  |  |
| No. of children (%) | -0 .035 | 0.254 |  |  |
| Hysterectomy (%) | 0.005 | 0.862 |  |  |
| Oophorectomy (%) | -0.015 | 0.578 |  |  |
| Current OCC use (%) | -0.121 | <0.001 |  |  |
| Current female hormone use (%) | 0.013 | 0.622 |  |  |

Abbreviations: BMI, body mass index; CVD, cardiovascular disease; hs-CRP, high-sensitive C-reactive protein; eGFR, estimated glomerular filtration rate; AST, aspartate aminotransferase; ALT, alanine aminotransferase; LDL, low-density lipoprotein; HDL, high-density lipoprotein; OCC, oral contraceptives.

**Supplementary Table S4**. Univariable and multivariable linear regression analyses for identification of variables associating with serum free thiol levels in postmenopausal women.

| **Variable** | **Univariable analysis** | | **Multivariable analysis** | |
| --- | --- | --- | --- | --- |
|  | St. Beta | *P*-value | St. Beta | *P*-value |
| Age (years) | -0.312 | <0.001 | -0.188 | <0.001 |
| BMI (kg/m^2^) | -0.181 | <0.001 | -0.107 | <0.001 |
| Waist circumference (cm) | -0.177 | <0.001 |  |  |
| Current smoking (%) | 0.107 | <0.001 |  |  |
| Alcohol use (%) | 0.099 | <0.001 |  |  |
| Systolic blood pressure (mmHg) | -0.132 | <0.001 |  |  |
| Diastolic blood pressure (mmHg) | 0.010 | 0.693 |  |  |
| CVD history (%) | -0.062 | 0.015 |  |  |
| Hypertension (%) | -0.132 | <0.001 |  |  |
| Diabetes (%) | 0.001 | 0.961 |  |  |
| Laboratory parameters |  |  |  |  |
| Hemoglobin (mmol/L) | 0.052 | 0.044 | 0.077 | 0.002 |
| hs-CRP (mg/L) | -0.094 | 0.001 |  |  |
| Albumin (g/L) | 0.110 | <0.001 | 0.057 | 0.026 |
| Creatinine (μmol/L) | -0.140 | <0.001 |  |  |
| eGFR (mL/min/1.73m^2^) | 0.301 | <0.001 | 0.168 | <0.001 |
| AST (U/L) | -0.082 | 0.001 | -0.066 | 0.008 |
| ALT (U/L) | -0.012 | 0.651 |  |  |
| Total cholesterol (mmol/L) | 0.023 | 0.373 |  |  |
| LDL-cholesterol (mmol/L) | -0.084 | 0.381 |  |  |
| HDL-cholesterol (mg/dL) | 0.061 | 0.020 |  |  |
| Triglycerides (mg/dL) | 0.046 | 0.078 |  |  |
| Glucose (mmol/L) | -0.042 | 0.105 |  |  |
| Gynecological variables |  |  |  |  |
| Pregnancy in past (%) | 0.014 | 0.601 |  |  |
| No. of children (%) | -0.130 | <0.001 |  |  |
| Hysterectomy (%) | 0.069 | 0.007 |  |  |
| Oophorectomy (%) | 0.029 | 0.256 |  |  |
| Current OCC use (%) | 0.033 | 0.201 |  |  |
| Current female hormone use (%) | 0.058 | 0.026 |  |  |

Abbreviations: BMI, body mass index; CVD, cardiovascular disease; hs-CRP, high-sensitive C-reactive protein; eGFR, estimated glomerular filtration rate; AST, aspartate aminotransferase; ALT, alanine aminotransferase; LDL, low-density lipoprotein; HDL, high-density lipoprotein; OCC, oral contraceptives.
